# Dysregulation of Translation Factors EIF2S1, EIF5A and EIF6 in Intestinal-Type Adenocarcinoma (ITAC)

**DOI:** 10.3390/cancers13225649

**Published:** 2021-11-11

**Authors:** Christoph Schatz, Susanne Sprung, Volker Schartinger, Helena Codina-Martínez, Matt Lechner, Mario Hermsen, Johannes Haybaeck

**Affiliations:** 1 Institute of Pathology, Neuropathology and Molecular Pathology, Medical University of Innsbruck, 6020 Innsbruck, Austria; christoph.schatz@i-med.ac.at (C.S.); susanne.sprung@i-med.ac.at (S.S.); 2 Institute of Otorhinolaryngology, Medical University of Innsbruck, Anichstrasse 35, 6020 Innsbruck, Austria; volker.schartinger@i-med.ac.at; 3 Department Head and Neck Oncology, Instituto de Investigación Sanitaria del Principado de Asturias, 33011 Oviedo, Spain; helenacm14@gmail.com (H.C.-M.); mariohermsen@gmail.com (M.H.); 4 UCL Cancer Institute, University College London, London WC1E 6AG, UK; m.lechner@ucl.ac.uk; 5 Barts Health NHS Trust, London E1 1BB, UK; 6 Diagnostic & Research Center for Molecular BioMedicine, Institute of Pathology, Medical University of Graz, 8036 Graz, Austria

**Keywords:** sinonasal adenocarcinoma of the intestinal type (ITAC), translation factors, biomarkers

## Abstract

**Simple Summary:**

Intestinal-type adenocarcinoma (ITAC) belongs to the group of sinonasal cancers which are a rare and heterogenous group of malignant neoplasms. Within this group, intestinal-type adenocarcinoma (ITAC) represents the most frequently occurring tumour, especially in Europe, and has been associated with exposure to occupational hazards, such as wood dust and leather. Eukaryotic translation initiation factors have been described as promising targets for novel cancer treatments, but hardly anything is known about these factors in ITAC. Here we performed in silico analyses, evaluated the protein levels of EIF2S1, EIF5A and EIF6 in tumour samples and non-neoplastic tissue controls obtained from 145 patients, and correlated these results with clinical outcome data, including tumour site, stage, adjuvant radiotherapy and survival. In silico analyses revealed significant upregulation of the translation factors EIF6 (ITGB4BP), EIF5, EIF2S1 and EIF2S2 (*p* < 0.05) with a higher arithmetic mean expression in ITAC compared to non-neoplastic tissue (NNT). Immunohistochemical analyses using antibodies against EIF2S1 and EIF6 confirmed a significantly different expression at the protein level (*p* < 0.05). In conclusion, this work identifies the eukaryotic translation initiation factors EIF2S1 and EIF6 to be significantly upregulated in ITAC. As these factors have been described as promising therapeutic targets in other cancers, this work identifies candidate therapeutic targets in this rare but often deadly cancer.

**Abstract:**

Intestinal-type adenocarcinoma (ITAC) is a rare cancer of the nasal cavity and paranasal sinuses that occurs sporadically or secondary to exposure to occupational hazards, such as wood dust and leather. Eukaryotic translation initiation factors have been described as promising targets for novel cancer treatments in many cancers, but hardly anything is known about these factors in ITAC. Here we performed in silico analyses, evaluated the protein levels of EIF2S1, EIF5A and EIF6 in tumour samples and non-neoplastic tissue controls obtained from 145 patients, and correlated these results with clinical outcome data, including tumour site, stage, adjuvant radiotherapy and survival. In silico analyses revealed significant upregulation of the translation factors EIF6 (ITGB4BP), EIF5, EIF2S1 and EIF2S2 (*p* < 0.05) with a higher arithmetic mean expression in ITAC compared to non-neoplastic tissue (NNT). Immunohistochemical analyses using antibodies against EIF2S1 and EIF6 confirmed a significantly different expression at the protein level (*p* < 0.05). In conclusion, this work identifies the eukaryotic translation initiation factors EIF2S1 and EIF6 to be significantly upregulated in ITAC. As these factors have been described as promising therapeutic targets in other cancers, this work identifies candidate therapeutic targets in this rare but often deadly cancer.

## 1. Introduction

Intestinal-type adenocarcinoma (ITAC) arises from cells within the nasal cavity and paranasal sinuses and belongs to the most frequently occurring sinonasal cancers in Europe and has been associated with exposure to occupational hazards, such as wood dust and leather [1,2,3,4,5]. ITACs most frequently occur in the ethmoid sinuses (40%), in the nasal cavity (25%) and in the maxillary antrum (20%) [3]. Men are more frequently affected at a mean age of 50–64 years [3].

Treatment for ITAC usually comprises endoscopic sinus surgery and adjuvant radiotherapy is indicated in advanced-stage and high-grade disease [1]. The overall 5-year survival (OS) ranges from 62 to 68.8% [6,7,8]. Compared to well-differentiated papillary ITACs, solid and mucinous subtypes have a poorer outcome [9,10,11]. Local recurrence usually occurs within 2 years of follow-up, although lymph node and distant metastases are infrequent [12]. Spread can occur to the skull base, intracranial space and to the orbit [3]. With regards to its immunophenotypic and histomorphologic characteristics ITAC is considered to be similar to colorectal adenocarcinomas [3,8].

Eukaryotic translation initiation factors have been described as promising targets for novel cancer treatments in many cancers, but hardly anything is known about these factors in ITAC. Hence, we focused on these translational factors and in view of a special expertise in our laboratory, we focused on EIF5A, EIF6 and EIF2S1, proteins mainly playing a role in the initiation phase of the translational process [13].

Moreover, our research particularly focused on EIF5A which is involved in the elongation of proteins [14,15] EIF6 in the nucleus [16] and in the cytoplasm with free 60S subunits [17,18] functions as a rate-limiting step of initiation [19,20] and acts as a ribosomal anti-association factor [21]. It prevents premature association with the 40S ribosomal subunit and is controlled by phosphorylation [22]. EIF2S1 plays a role in the recruitment of Met-tRNA_i_ to the 40S/mRNA complex, non-AUG initiation and re-initiation, important for the translational control of specific mRNAs [23].

Changes at protein level of these eukaryotic translation initiation factors has been shown to lead to uncontrolled cell growth and has been implicated in carcinogenesis and the progression of disease [24,25,26].

The aim of this study was to identify eukaryotic translation initiation factors that may be dysregulated and therefore represent candidate therapeutic targets.

## 2. Materials and Methods

### 2.1. In silico Analysis of Publicly Available Data on Adenocarcinoma

**Figure 1:**
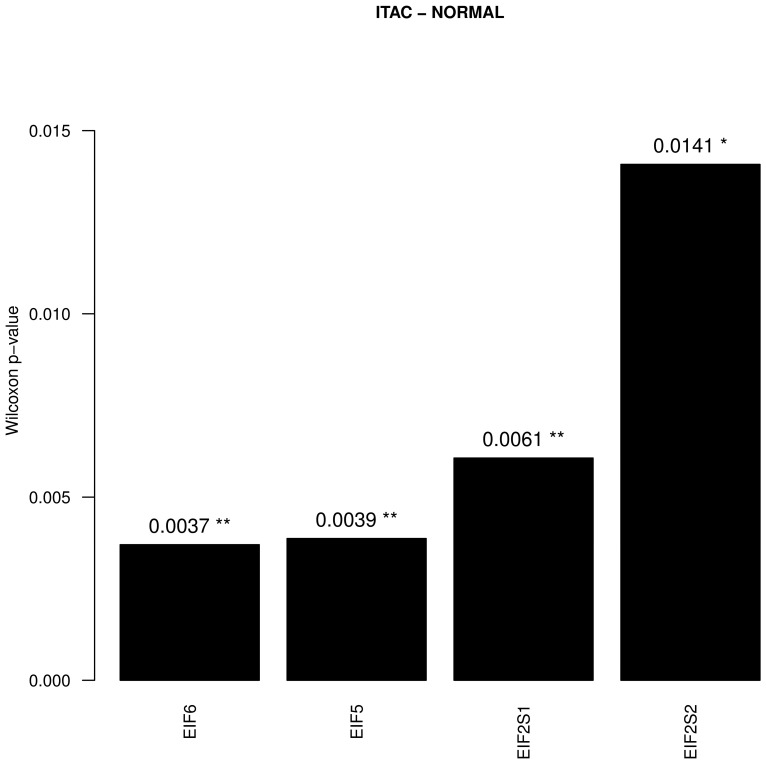
Significant Wilcoxon *p*-values of the translation factors (*p* < 0.05). Each factor was compared against the same factor between the groups ITAC and NNT in the dataset GSE17433. Black bars indicate that the arithmetic mean of the first group (ITAC) was higher than the arithmetic mean of the second group (NNT) and white bars indicate the opposite. ** indicate a *p*-value < 0.01, * indicates a *p*-value < 0.05.

The publicly available dataset GSE17433 was analysed. This dataset contains 18 samples in total, including 8 ITAC samples, 9 non-neoplastic (NNT) samples and 1 colloid adenocarcinoma sample. ITAC and non-neoplastic samples were compared against each other (Figure 1). The colloid adenocarcinoma sample was excluded from analysis. Cancer samples were compared with controls in order to identify differences in gene expression using the R function wilcox.test with a significance threshold of *p* < 0.05. Eukaryotic initiation translation, elongation translation and releasing factors were identified and the data was further processed via a C# script with use of the REngine to identify the most differentially expressed translation factors.

### 2.2. Analysis and Validation of Significant eIFs In Vitro

**Figure 2:**
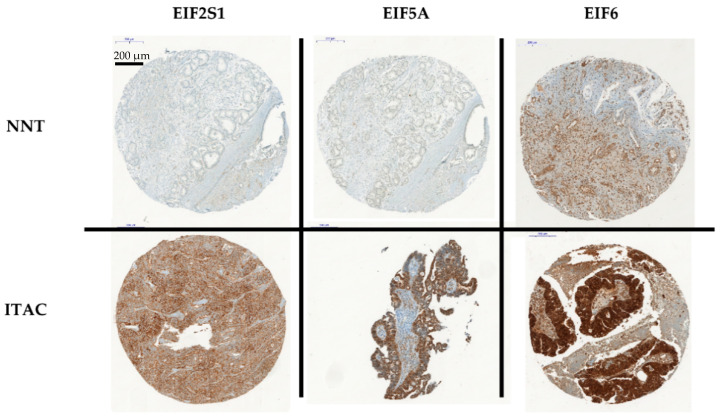
NNT (**upper row**), ITAC (**lower row**). Stained tissue sections using antibodies against EIF2S1, EIF5A and EIF6 in tissue micro arrays (TMAs) at different resolutions.

In order to validate the above results, tissue samples of 145 ITACs were subjected to immunohistochemical (IHC) analysis using an optimized protocol with antibodies against EIF2S1, EIF5A and EIF6 based on the prior in silico analysis (Figure 2).

Regarding the category ‘RecMet (Recurrence or Metastases) for gene upper-RecMet gene lower’ the R function ‘survcorr’ for the correlation was used, as well as ‘pchisq’ for calculating the *p*-value. The R functions ‘SurvCorr’ and ‘surv’ from the package ‘survival’ were also utilized.

**Figure 3:**
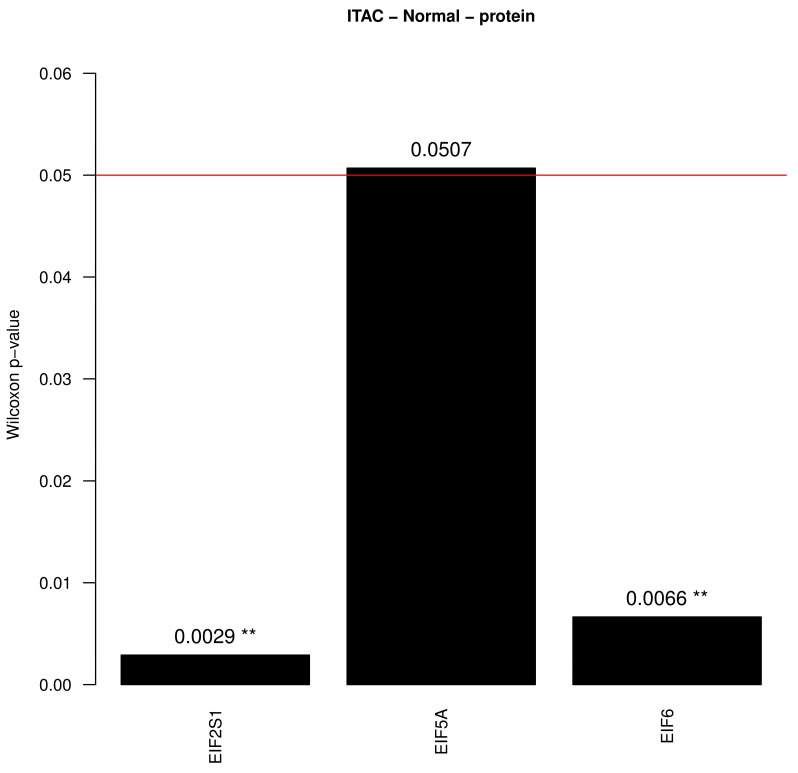
Significant Wilcoxon *p*-values of the translation factors (*p* < 0.05) between the groups ITAC and NNT in TMA samples. Black bars and uppercase letters indicate that the arithmetic mean of the scores of the first group (ITAC) was higher than the arithmetic mean of the scores of the second group (NNT) and white bars and lowercase letters indicate the opposite. ** indicates a *p*-value < 0.01. The horizontal red line marks the threshold of 0.05.

For the immunohistochemical (IHC) validation tissue micro arrays (TMAs) with cancer and normal mucosa samples (2 to 3 replicates per patient) were obtained and stained. The Quick score was calculated by multiplication of the intensity and density, as described previously [27] (Figure 3). The highest value for each patient was selected in ITAC and in NNT. Supplementary Figure S1 shows Intensities for EIF2S1, EIF5A and EIF6 exemplary. 

Based on the scores, Spearman’s rho was calculated using the R function ‘cor’ and additionally ‘cor-test’ to obtain the *p*-value.

Additionally the groups ‘longer RecMet-shorter RecMet’ and ‘RecMet gene upper-RecMet gene lower’ were built. The median was used as cut-off.

The values of each translation factor were compared against the values of the same translation factor from other categories using the R function wilcox.test to calculate the Wilcoxon *p*-value for a comparison of distribution differences.

**Figure 4:**
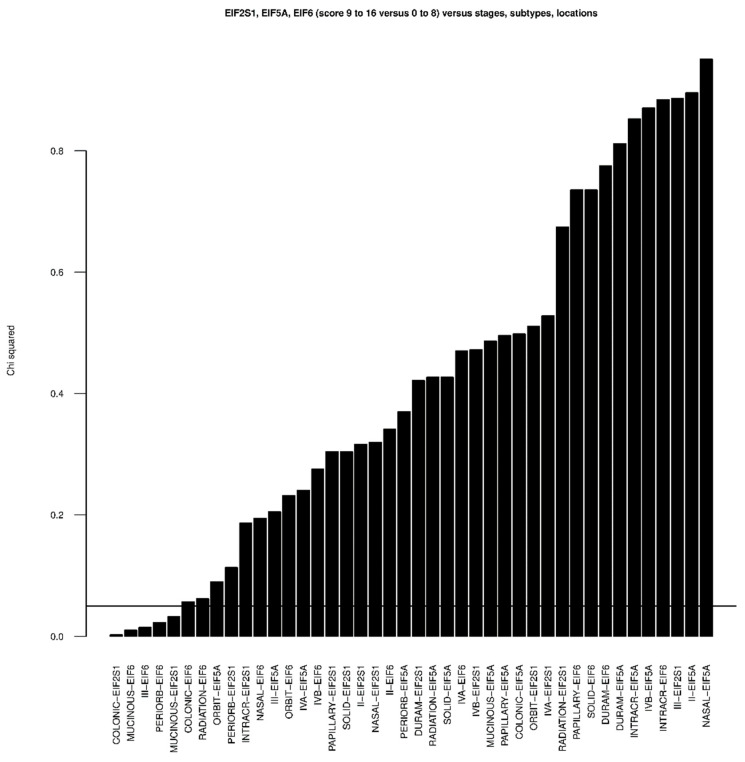
Chi^2^ *p*-values based on the scores for the proteins EIF2S1, EIF5A and EIF6 in combination with each subtype, location, stage and radiotherapy. Scores of 9 to 16 were considered as ‘yes’, scores of 0 and 8 as ‘no’. The horizontal line marks the threshold of 0.05.

For each of the translation factors EIF2S1, EIF5A and EIF6, a χ^2^ analysis was performed regarding locations, subtypes, stages and radiotherapy whereas scores of 0 to 8 were considered as ‘no’ and scores of 9 to 16 were considered as ‘yes’ depending on the specific location, subtype, stage and radiotherapy (Figure 4). The R function ‘chisq.test’ was used.

A score was calculated regarding the density and intensity for each translation factor of the TMA (tissue micro array). Groups were built based on clinical information ‘subtype’, ‘stage’ ‘intracr’ (intracranial), ‘duram’ (duramater), ‘orbit’, ‘periorb’, ‘nasal’, ‘wood’, ‘yearswood’, ‘tobacco exposure’, ‘timetorecmet’ (time to recurrence or metastasis = disease-free survival), ‘radiotherapy’, ‘met’ (Metastases) and ‘Exit’ (Table 1).

### 2.3. Ethics Committee

The sample collection was approved by the Institutional Ethics Committee of the Hospital Universitario Central de Asturias and by the Regional CEIC from Principado de Asturias (approval numbers: 83/17 for project PI17/00763 and 07/16 for project CICPF16008HERM).

### 2.4. Immunohistochemistry

Immunohistochemistry was performed on sections of each TMA. Primary eIF6 antibody (rabbit polyclonal A303-030A-M, Bethyl/Biomol, Montgomery, AL, USA), primary EIF5A (rabbit, polyclonal PA5-29204, Invitrogen, Carlsbad, Germany) and primary EIF2S1 (rabbit D7D3 5324, monoclonal, Cell Signaling, Danvers, MA, USA) were stained on Ventana Immunostainer BenchMark (Roche, Karlsruhe, Germany) using ultra-VIEW Universal DAB Detection Kit (Ventana Medical Systems, Tucson, AZ, USA) and mCC1/sCC1.

## 3. Results

Samples from 145 previously untreated ITAC patients treated between 1978 and 2014 were collected from the biobank archives of the Hospital Universitario Central de Asturias. All patients had signed an informed consent for the collection, analysis and storage of their biological material, and the study was approved by the Institutional Ethics Committee of the Hospital Universitario Central de Asturias and by the Regional CEIC from Principado de Asturias (approval number 07/16 for project CICPF16008HERM). The mean patient age was 66 years, and 143 (143/145) of patients were males. The distribution of disease stage according to the TNM system for tumour classification [28] was: 31 with tumour stage I, 17 with stage II, 48 with stage III, 17 with stage IVa and 26 with stage IVb. A total of 13 cases were papillary, 88 were colonic, 10 were solid and 13 were of the mucinous histological subtype [8]. A history of professional exposure to wood dust was recorded for 123 of 145 (88.49%) patients. A total of 81 (88.27%) patients received radiotherapy after radical surgery. The median follow-up was 60 months (range 0–460). Details on the clinical features are presented in Table 1.

At the level of gene expression a comparison between ITAC and NNT in the dataset GSE17433 revealed a significantly higher expression of the translation factors *EIF6*, *EIF5, EIF2S1* and *EIF2S2* in ITAC (Figure 1, Supplementary Figures S5 and S6). Overall, 36 initiation and elongation translation factors were tested for expression differences between NNT and ITAC samples.

TMAs of ITAC and NNT were then stained for antibodies for EIF2S1, EIF5A and EIF6 (Figure 2). After comparison and calculation of the score, significantly higher protein levels of the translation factors EIF2S1, EIF5A and EIF6 were confirmed by immunohistochemistry, based on a higher arithmetic mean score in ITAC (Figure 3). Work on EIF5A encompassed 119 samples of ITAC and 8 samples of NNT, work on EIF6 encompassed 120 samples of ITAC and 6 samples of NNT and work on EIF2S1 encompassed 118 samples of ITAC and 8 samples of NNT (Supplementary Table S1, Supplementary Figures S3 and S4).

**Figure 5:**
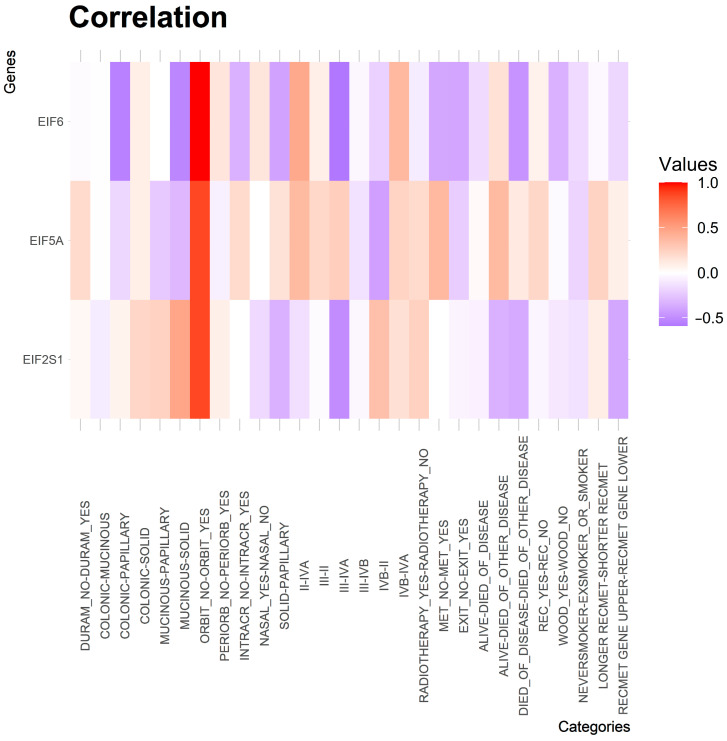
Spearman correlations between groups and the translation factors EIF2S1, EIF5A and EIF6. The groups are based on clinical information. The scores of each eIF from the first group was correlated with the scores of the second group. For ‘RecMet gene upper-RecMet gene lower’ the recurrence time of the group with the higher scores was used (median split) and was correlated with the group with the lower scores for each eIF.

Spearman correlations between each EIFs in ITAC and clinical variables are shown in Figure 5. EIF2S1 showed a strong negative correlation (rho < −0.5) between stage III and stage IVA (Wilcoxon *p*-value = 0.3353) and a moderate correlation (rho < −0.3) between the upper–lower dichotomization of time to recurrence or metastasis (*p* > 0.0000). Trends (rho < −0.1) were observed for presence and absence in the nasal region (*p* = 0.3604), between stage II and IVA (*p* = 0.3353), between never smoker and ex-smoker or smoker (*p* = 0.2191) and between exposure to hardwood dust and no exposure (*p* = 0.7766) (Figure 5, Supplementary Figure S2).

EIF6 revealed a high negative correlation (rho < −0.4) between stage III and stage IVA (Wilcoxon *p*-value = 0.1425), between died of disease versus died of other disease (*p* = 0.8980) and between the subtypes colonic and papillary, between mucinous and solid (*p* = 0.2273) and between solid versus papillary. Moderate anticorrelations (rho < −0.2) were obtained after comparisons between no occurrence of death and between the occurrence of death (*p* = 0.4562), between the presence and the absence of metastases (*p* = 0.9934), between a spread to the intercranial space and no spread to the intercranial space (*p* = 0.7835) and between an exposure to hardwood dust and no exposure (*p* = 0.8227). Mild anticorrelations (rho < −0.1) were found between stage IVB and stage II (*p* = 0.5818), between an upper–lower dichotomization of time to recurrence or metastasis (*p* < 0.000), between never smoker and ex-smoker or smokers (*p* = 0.8980) and overall survival (*p* = 0.5361) (Figure 5, Supplementary Figure S2).

Strong negative correlations (rho < −0.4) regarding EIF5A were retrieved after a comparison between stage IVB and stage II (Wilcoxon *p*-value = 0.6804). Moderate negative correlations (rho < −0.2) were found between the subtypes mucinous and solid (*p* = 0.6644) and between mucinous and papillary (*p* = 0.3250) and between the occurrence of death and no occurrence of death (*p* = 0.5000). Analyses between never smoker versus ex-smoker or smoker (*p* = 0.2258), between the subtype colonic versus papillary (*p* = 0.5476) and stage III versus stage IV (*p* = 0.8727) revealed mild negative correlations (rho < −0.1) (Figure 5, Supplementary Figure S2).

*p*-values below 0.05 were obtained for EIF6 and the subtype mucinous, for the periorbital extension and for stage III. A *p*-value below 0.05 was calculated for EIF2S1 and the subtype colonic and the subtype mucinous (Figure 4).

However, most of these correlations were not statistically significant and a final conclusion cannot be drawn.

**Figure 6:**
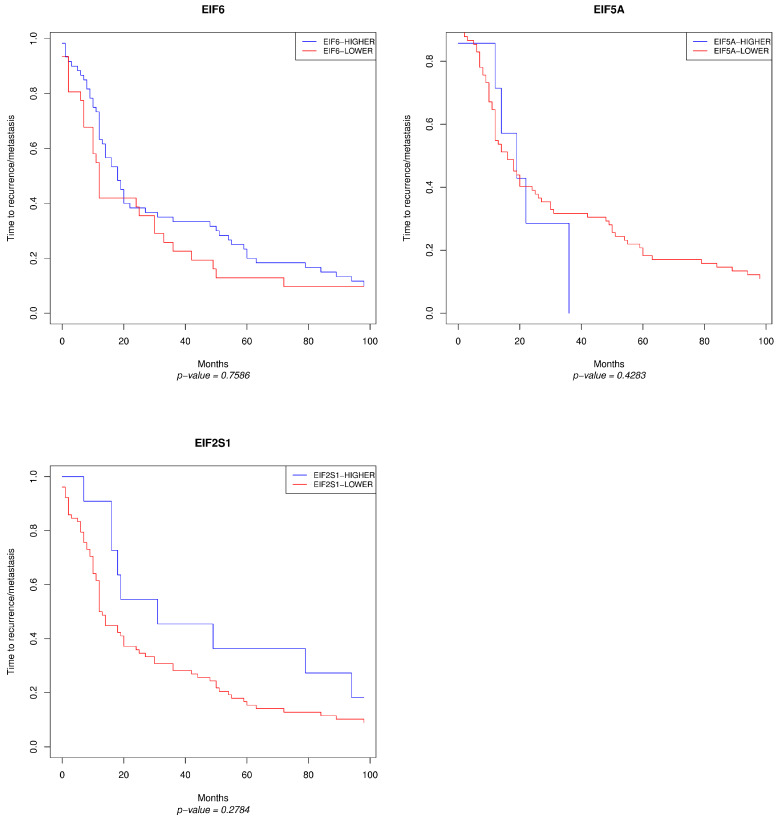
Kaplan–Meier diagrams of EIF2S1, EIF5A and EIF6 with scores of 1, 2, 3, 4 and 8 combined to ‘Lower’ and scores of 12 and 16 combined to ‘Higher’ based on time to recurrence/metastasis. The y-axis shows the survival probability, the x-axis the duration in months.

Higher density and intensity scores of EIF6 and EIF2S1 each revealed a difference in the disease-free survival (recurrence or metastasis), without a significant *p*-value as shown in Figure 6. Besides this result with the Quick score and ITAC samples, higher gene expression of EIF6 is associated with tumour progression in some cancer types [29], especially in colorectal cancer [26] and in colon cancer [26], in lung metastases [30], in acute promyelocytic leukaemia [31], in ovarian serous carcinoma [32] and in malignant mesothelioma [33].

Time to recurrence or metastases associated with EIF2S1 score 8 versus score 12, score 8 versus score 3, score 8 versus score 4, score 3 versus score 12, score 3 versus score 4 and score 4 versus score 12 revealed a hazard ratio (HR) higher than 1 (Supplementary Table S2). Time to recurrence or metastases of EIF5A score 12 versus score 8, score 1 versus score 8, score 4 versus score 8, score 1 versus score 12, score 4 versus score 12 and score 1 versus score 4 showed a hazard ratio higher than 1 (Supplementary Table S2). Time to recurrence or metastases associated with EIF6 score 16 compared to score 8 and score 16 compared to score 12 revealed a hazard ratio higher than 5. Score 16 versus score 4 revealed a hazard ratio higher than 3. Score 4 compared to score 8, score 12 compared to score 4 and score 8 compared to score 12 led to a hazard ratio higher than 1 (Supplementary Table S2). Time to recurrence or metastases associated with EIF5A compared to EIF2S1 regarding a score of 12 revealed a hazard ratio higher than 2 (Supplementary Table S3). Time to recurrence or metastases associated with EIF5A compared to EIF2S1 regarding a score of 4 and EIF5A compared to EIF6 regarding a score of 12 showed a hazard ratio higher 1 (Supplementary Table S3). Time to recurrence or metastases associated with EIF2S1 compared to EIF5A regarding a score of 8 led to a hazard ratio higher than 1 (Supplementary Table S3). Regarding a score of 12 and a score of 4 depending on time to recurrence or metastases, EIF6 versus EIF2S1 and EIF6 versus EIF5A regarding a score of 8 depending on time to recurrence or metastases revealed a hazard ratio higher than 1 (Supplementary Table S3). EIF5A versus EIF6 regarding a score of 4 depending on the time to recurrence or metastases led to a hazard ratio higher than 1 (Supplementary Table S3).

## 4. Discussion

Translation factors play a central role in the initiation phase of the translation of mRNA in eukaryotic cells and hence determine the cellular phenotype. mRNA translation has been described as a multifactorial process, influenced and regulated by many factors and governed through complex intracellular pathways and influenced by the cellular microenvironment. Changes in the levels of proteins that govern translation can alter cell characteristics and also play a role in carcinogenesis or resistance to anticancer treatment. In eukaryotes, eIFs including eIF1, eIF1A, eIF3 and eIF5 form a complex that is involved in the shift from initiation to elongation. Cellular pathways which have also been implicated in tumorigenesis and the cell-cycle, such as MAPK and PI3K/AKT/mTOR, are able to regulate and promote functions of eIFs [34] and therapeutic targeting of these pathways also have an effect on eIFs [35,36]. Hence, the dysregulation of eIFs and eEFs has been demonstrated in many human cancers, e.g., EIF2S1 was shown to resist cell death during paclitaxel treatment of cells [37]. eIF5A is one of the key proteins of translation initiation. On the one hand it works as a GAP (GTPase-activating protein), on the other hand as an inhibitor of eIF2B. EIF5A was found to be dysregulated in high-grade colon and in rectal carcinoma [26] and in several other tumour types, such as glioblastoma, cervical and ovarian cancer, bladder cancer and non-small-cell lung cancer [29]. Lastly, eIF6 is a rate-limiting factor in translational regulation and it is able to block the interaction of the 40S and 60S subunit [29]. Furthermore its dysregulation by overexpression has been described in low- and high-grade colon and rectal carcinoma [26], pancreatic ductal adenocarcinoma [24], in gallbladder cancer [38], in non-small cell lung cancer [38], in head and neck cancer and in acute promyelocytic leukaemia [29]. Hence, it is not a surprise that these factors have also been found to be dysregulated in ITAC, both in the course of the analysis of publicly available datasets and in the course of the extensive immunohistochemical dataset of a large group of ITAC patients.

Taking into account all investigated eIFs, eIF6 appears to play the most important role in ITACs, followed by eIF2S1, with increased expression in tumour tissues compared with normal controls. Lower levels of eIF2S1 appear to be associated with worse disease-free survival and this is interesting with regards to its potential as a prognostic biomarker.

Of course, this is only a first investigation into the role of these factors in ITAC and more studies and larger datasets are needed to confirm the findings, but with novel therapies being developed that target these factors in other cancers, this work provides a great starting point and direction for further studies.

## 5. Conclusions

This work identifies the eukaryotic translation initiation factors EIF2S1 and EIF6 to be significantly upregulated in ITAC. As these factors have been described as promising therapeutic targets in other cancers, this work identifies candidate therapeutic targets in this rare but often deadly cancer.

## Figures and Tables

**Table 1:** Clinical data of patients’ tissues. For control NNT from the patients were used.

Variables	Tissues for Biochemical Analyses (*n* = 145)
Age (Median)	66 years
Sex	
Male	143 (98.62%)
Female	2 (1.38%)
Subtype	
Papillary	13 (8.97%)
Colonic	88 (60.69%)
Solid	10 (6.9%)
Mucinous	13 (8.97%)
Stage	
Stage I	31 (22.3%)
Stage II	17 (12.33%)
Stage III	48 (34.53%)
Stage IVA	17 (12.33%)
Stage IVB	26 (18.71%)
Intracranial spread	
No	123 (88.49%)
Yes	16 (11.51%)
Dural infiltration	
No	114 (82.01%)
Yes	25 (17.99%)
Orbital extension	
No	135 (97.12%)
Yes	4 (2.88%)
Periorbital extension	
No	120 (86.33%)
Yes	19 (13.67%)
Nasal cavity only	
No	55 (39.86)
Yes	83 (60.14%)
Exposure to wood dust	
No	16 (11.51%)
Yes	123 (88.49%)
Years exposure to wood dust (Median)	35
Tobacco exposure	
Never smoked	61 (46.92%)
Formal smoker or smoker	69 (53.08%)
Months of follow-up (Median)	60
Months to recurrence or metastasis (Median)	16
Adjuvant radiotherapy	
No	58 (41.73%)
Yes	81 (58.27%)
Recurrence	
No	75 (53.96%)
Yes	64 (46.04%)
Metastases	
No	123 (88.49%)
Yes	16 (11.51%)
Status	
Alive	60 (43.17%)
Died of disease	58 (41.73%)
Died of other cause	21 (15.11%)

## Data Availability

Exclude this statement as the study did not report any data.

## Supplementary Materials

The following are available online at https://www.mdpi.com/article/10.3390/cancers13225649/s1, Figure S1: Intensity ranges from 1 to 3 of EIF2S1, EIF5A and EIF6. Figure S2: Wilcoxon *p*-values clinical data with translation factors. Figure S3: Histograms of ITACs, respectively, NNT and EIF5A, EIF6 and EIF2S1. Figure S4: Boxplot of ITACs, respectively, NNT and EIF5A, EIF6 and EIF2S1. Figure S5: Heatmap of EIF5, EIF2S2 and EIF2S1 based on each sample of the GSE17433 dataset. Figure S6: Heatmap of EIF5, EIF2S2 and EIF2S1 based on average values of the samples of the GSE17433 dataset. Supplementary Table S1: Number of samples with scores. Supplementary Table S2: Hazard ratios of comparisons between scores of EIF2S1, EIF5A and EIF6. Supplementary Table S3: Hazard ratios of comparisons between EIF2S1, EIF5A and EIF6 depending on the same score.
