# Supplementary material for: Dysregulation of Translation Factors EIF2S1, EIF5A and EIF6 in Intestinal-Type Adenocarcinoma (ITAC)
Source: Cancers (Basel). 2021 Nov 11;13(22):5649. doi: 10.3390/cancers13225649 (PMC8616251; doi:10.3390/cancers13225649)
Supplement: Supplementary file 1 [file cancers-13-05649-s001.zip › cancers-1451397-supplementary.pdf]

cancers

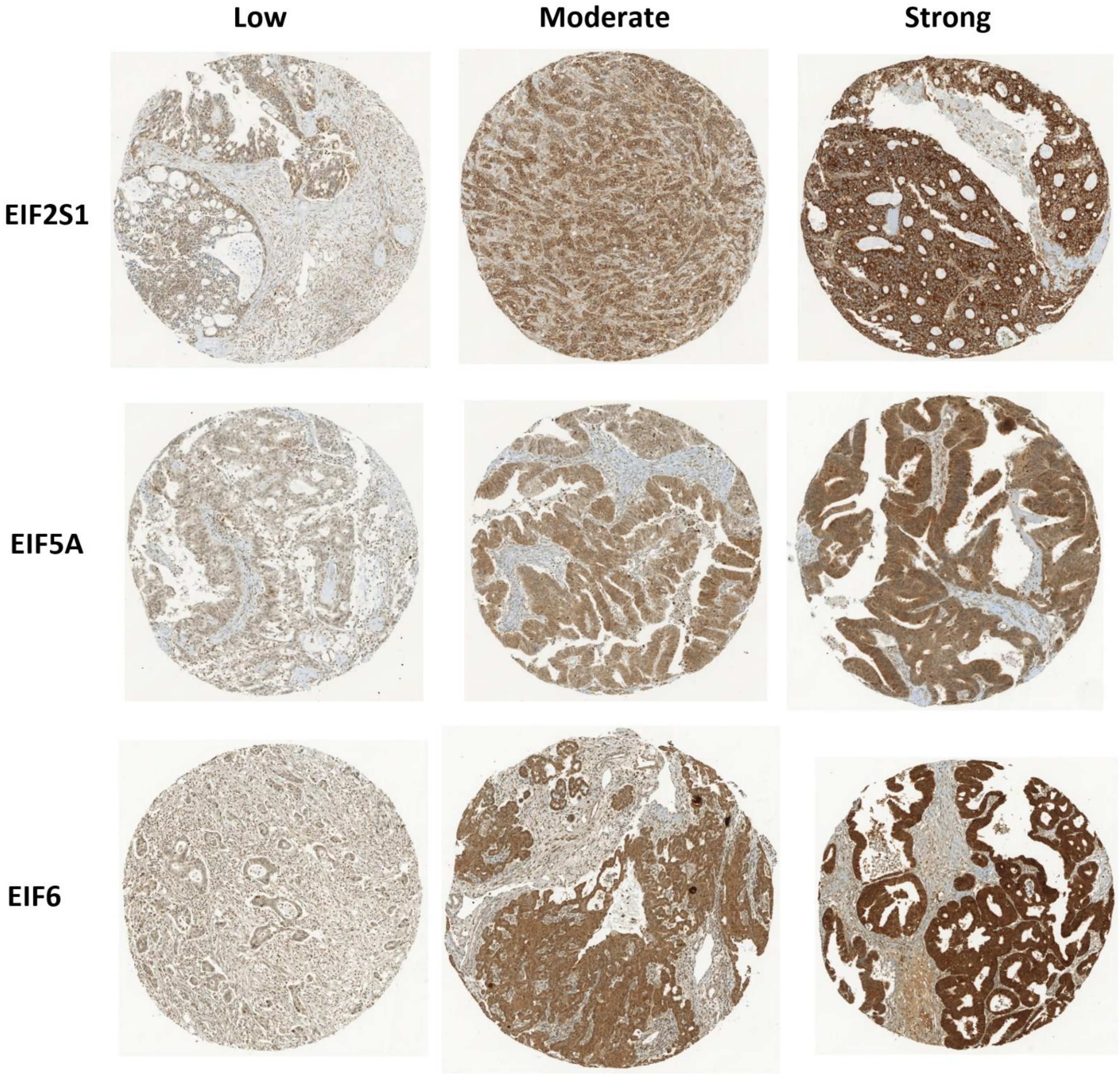

Figure S1. Antibody treated Tissue Micro Arrays from Intensity 1 to 3 for EIF2S1, EIF5A and EIF6.

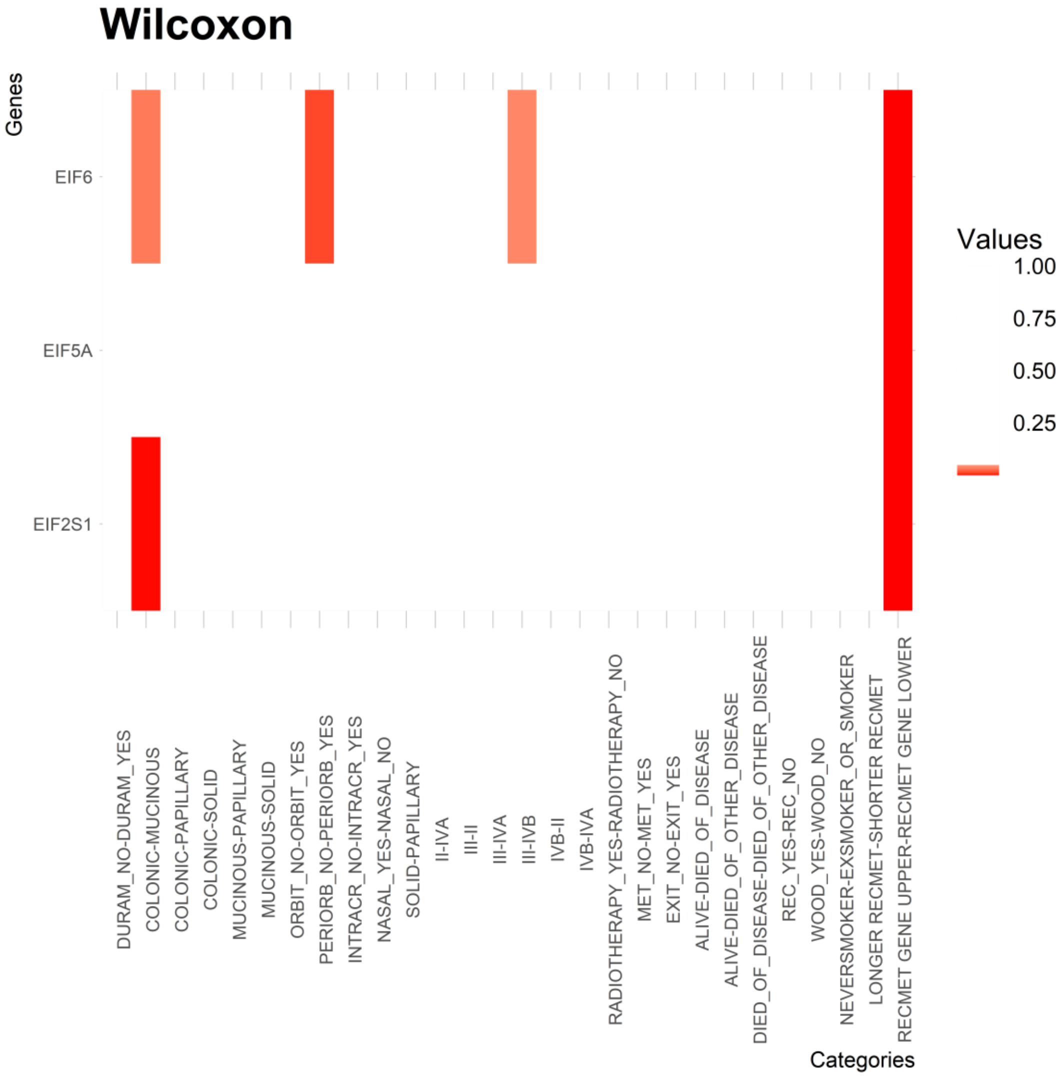

Figure S2. Wilcoxon p-values based on the scores for the proteins EIF2S1, EIF5A and EIF6 between the groups. The groups were built based on clinical information.

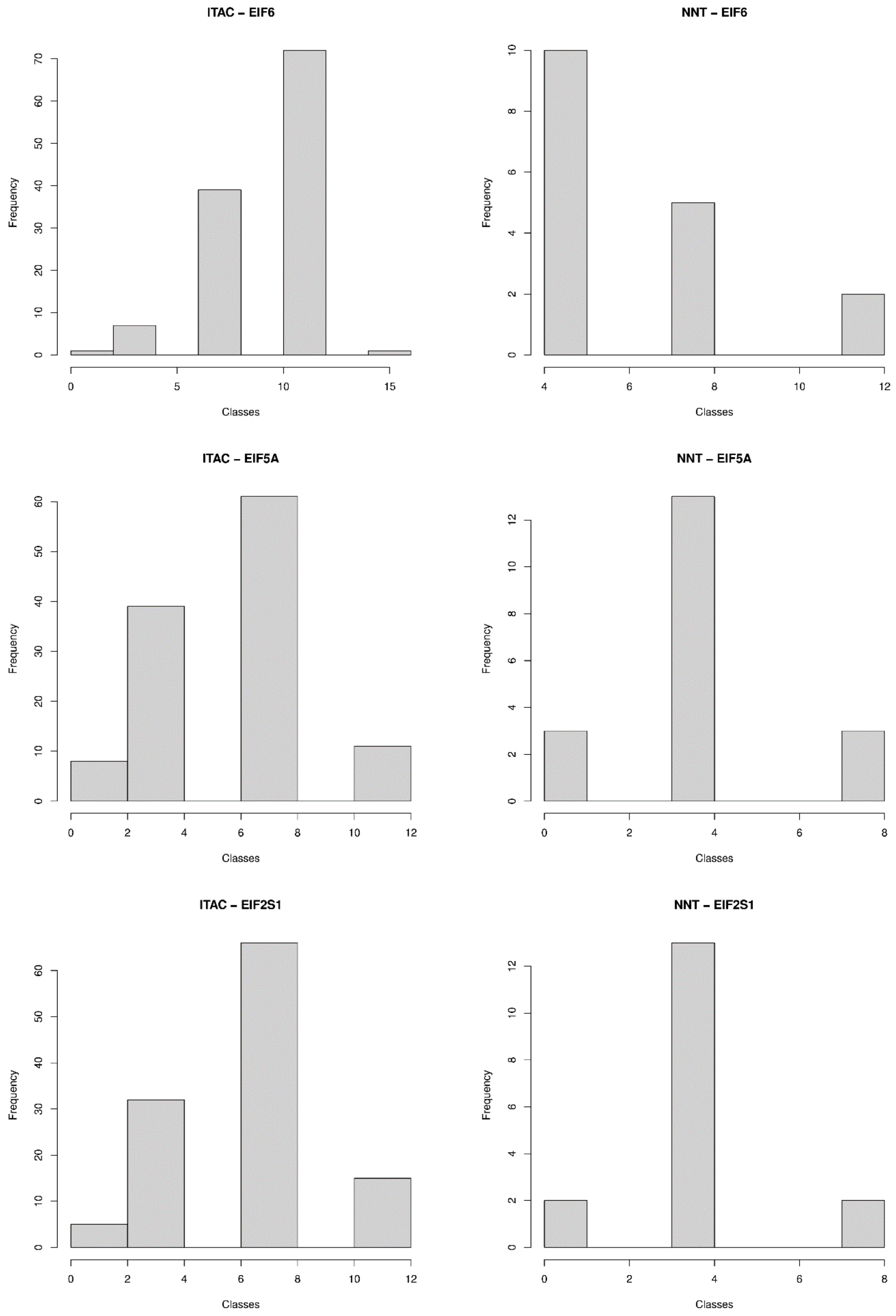

Figure S3. Histograms of ITACs respectively NNT and EIF5A, EIF6 and EIF2S1. On the x-axis the score and on the y-axis the frequency are applied.

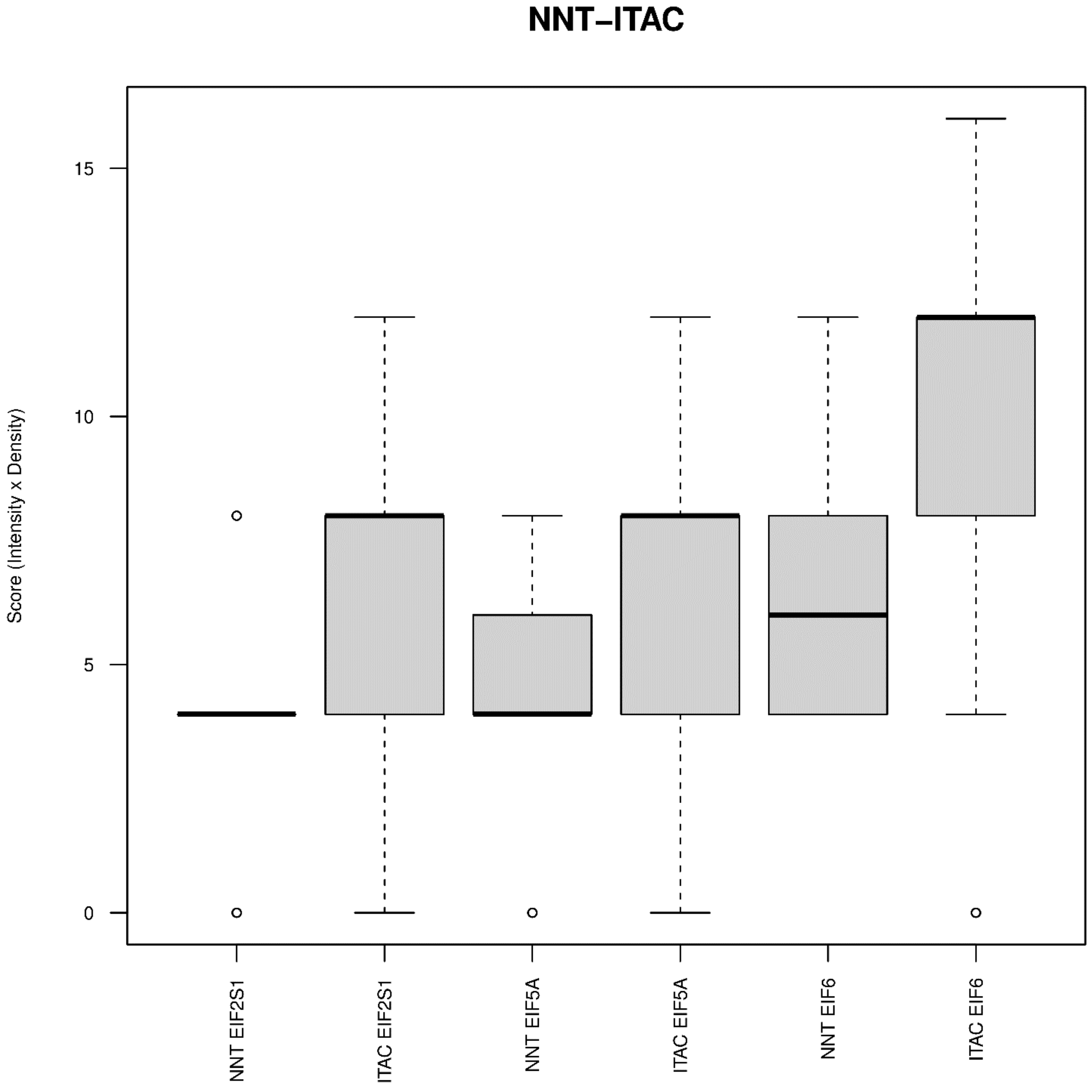

Figure S4. Boxplot of ITACs respectively NNT and EIF5A, EIF6 and EIF2S1.

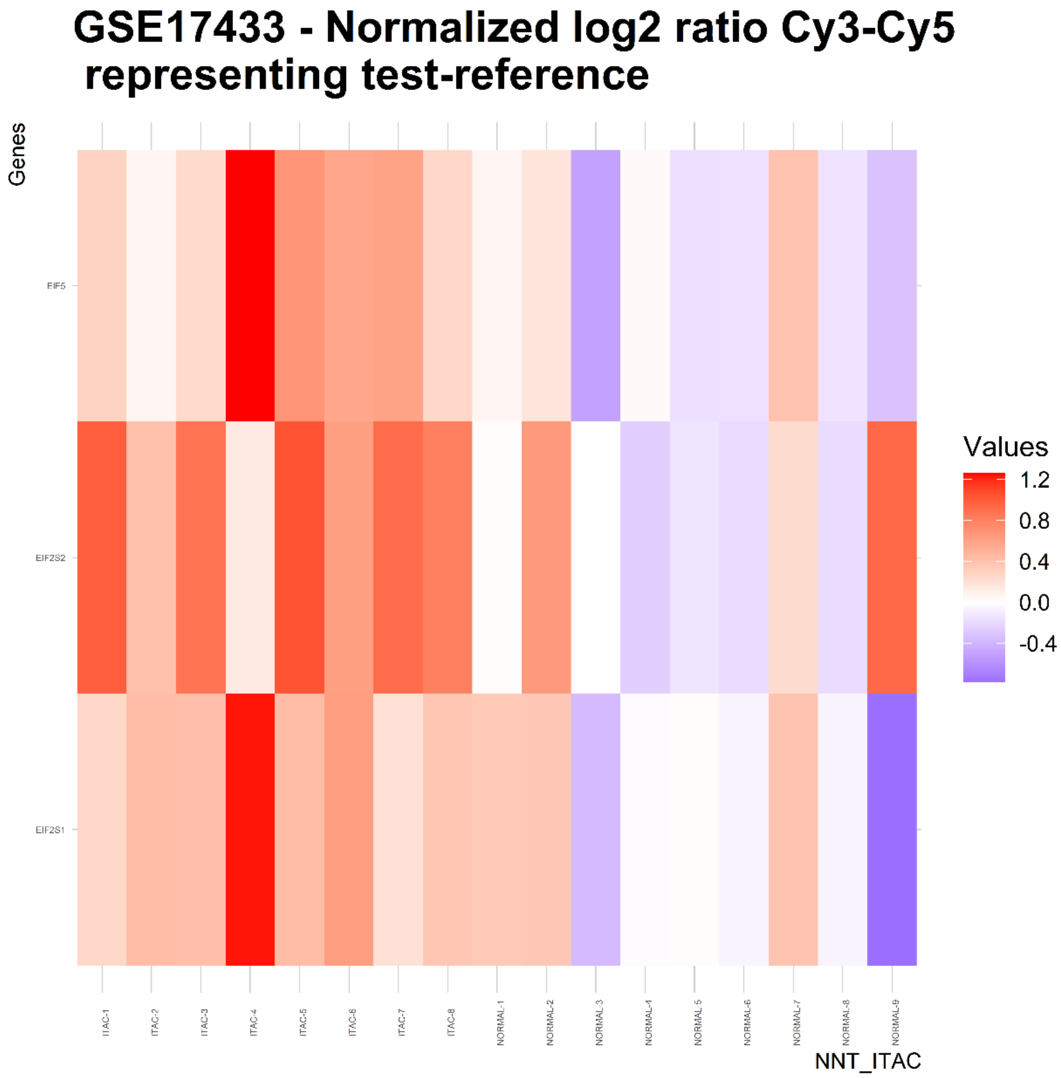

Figure S5. Heatmap of EIF5, EIF2S2 and EIF2S1 based on each sample of the GSE17433 dataset.

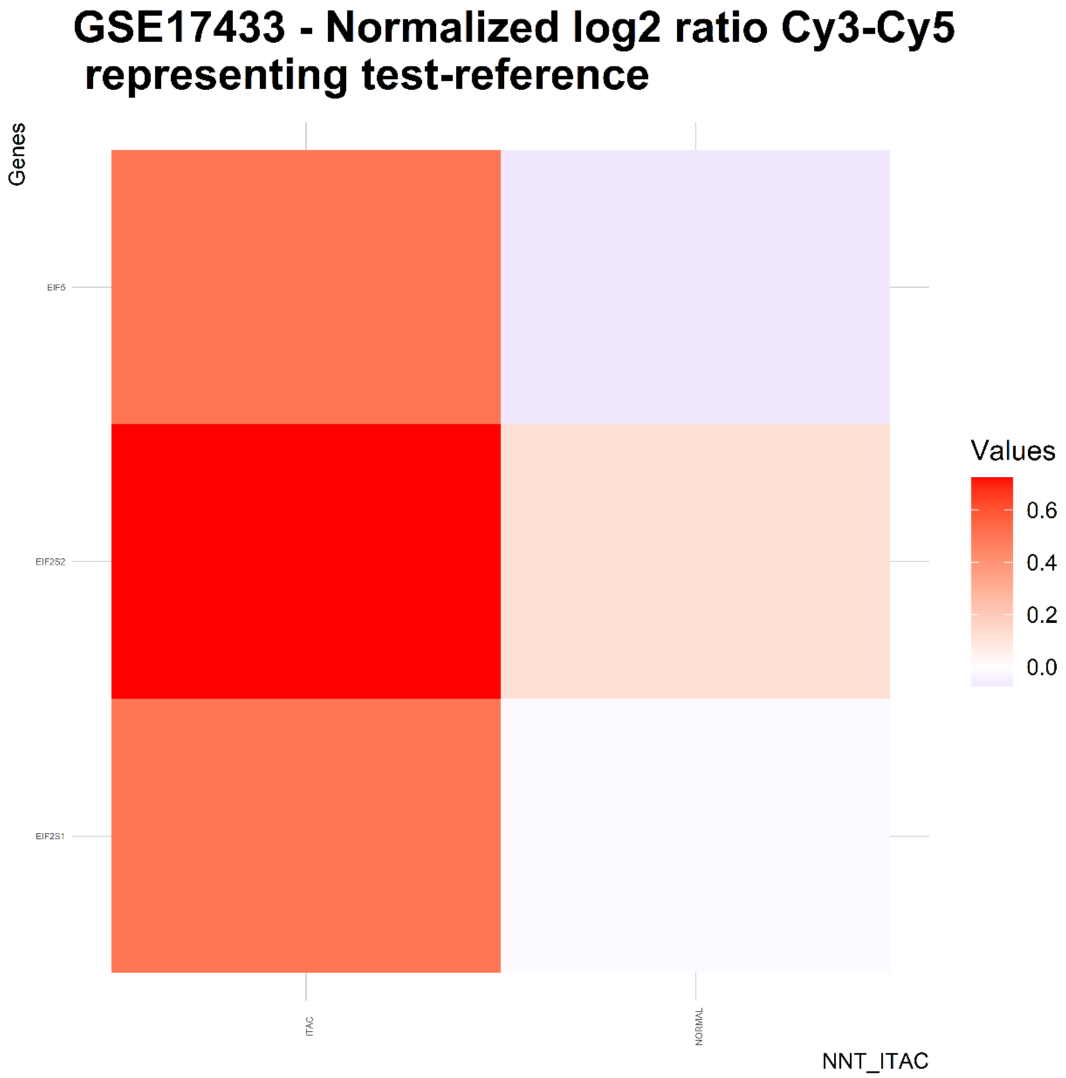

Figure S6. Heatmap of EIF5, EIF2S2 and EIF2S1 based on average values of the samples of the GSE17433 dataset.

Table S1. Number of samples for NNT and ITAC.

	NNT	*ITAC*
EIF5A	*8*	*119*
EIF6	*6*	*120*
EIF2S1	*8*	*118*

Table S2. Hazard Ratios of comparisons between scores of EIF2S1, EIF5A and EIF6.

Translation Factor	Score versus	Score	Hazard Ratio
EIF2S1	8	12	1.5646
EIF2S1	8	3	1.4047
EIF2S1	8	4	1.3731
EIF2S1	3	12	1.2546
EIF2S1	3	4	1.1823
EIF2S1	4	12	1.0364
EIF2S1	12	4	0.9649
EIF2S1	4	3	0.8458
EIF2S1	12	3	0.7971
EIF2S1	4	8	0.7283
EIF2S1	3	8	0.7119
EIF2S1	12	8	0.6391
EIF5A	12	8	1.7623
EIF5A	1	8	1.7359
EIF5A	4	8	1.5294
EIF5A	1	12	1.3573
EIF5A	4	12	1.1088
EIF5A	1	4	1.0887
EIF5A	4	1	0.9186
EIF5A	12	4	0.9019
EIF5A	12	1	0.7368
EIF5A	8	4	0.6538
EIF5A	8	1	0.5761
EIF5A	8	12	0.5674
EIF6	16	8	> 5.0000
EIF6	16	12	> 5.0000
EIF6	16	4	3.6026
EIF6	4	8	1.2414
EIF6	12	4	1.1105
EIF6	8	12	1,0622
EIF6	12	8	0.9415
EIF6	4	12	0.9005
EIF6	8	4	0.8056
EIF6	4	16	0.2776
EIF6	12	16	0.0665
EIF6	8	16	0.0385

Table S3. Hazard Ratios of comparisons between EIF2S1, EIF5A and EIF6 depending on the same score.

Translation Factor versus	Translation Factor	Score	Hazard Ratio
EIF5A	EIF2S1	12	2.0671
EIF5A	EIF2S1	4	1.5873
EIF5A	EIF6	12	1.4604
EIF2S1	EIF5A	8	1.3509
EIF6	EIF2S1	12	1.3085
EIF6	EIF2S1	4	1.3077
EIF5A	EIF6	4	1.2156
EIF6	EIF5A	8	1.2145
EIF2S1	EIF6	8	1.0953
EIF6	EIF2S1	8	0.9130
EIF5A	EIF6	8	0.8234
EIF6	EIF5A	4	0.8226
EIF2S1	EIF6	4	0.7647
EIF2S1	EIF6	12	0.7642
EIF5A	EIF2S1	8	0.7403
EIF6	EIF5A	12	0.6848
EIF2S1	EIF5A	4	0.6300
EIF2S1	EIF5A	12	0.4838
